# Patient Perspective on Medical Conditions Post Immune Checkpoint Therapy in Advanced Renal Carcinoma

**DOI:** 10.15586/jkc.v12i2.383

**Published:** 2025-04-03

**Authors:** Ulka Vaishampayan, Sumanta Pal, Sephora Dafinescu, Neha Shah, Dena Battle, Michael Staehler

**Affiliations:** 1 Medicine/Oncology, Rogel Cancer Center University of Michigan Ann Arbor MI;; 2 Oncology City of Hope, Duarte CA, Rogel Cancer Center University of MI;; 3 Research Coordinator, Rogel Cancer Center University of Michigan, MI;; 4 President, KCCure, Kidney Cancer Research Alliance, Washington DC-Baltimore Area;; 5 Urology, University of Munich Germany

**Keywords:** patient reported outcomes, chronic toxicities, immunotherapy, kidney cancer

## Abstract

Immune checkpoint therapy (ICI) has enabled induction of remission in advanced renal cell carcinoma RCC. ICI toxicities can persist as chronic health conditions. We developed a patient survey to assess changes in medical comorbidities after ICI. The survey was developed by the Kidney Cancer Research Alliance (KCCure), with multidisciplinary representation from urologic surgeons, medical oncologists, and patient advocates. The survey was broadcast between July 2022 and September 2022 to patients via website, mailing lists, and social media platforms. Patient perspective on changes to any medical conditions were evaluated in the survey questionnaire. Of 1062 patients that responded, 399 were self-identified as being metastatic and 289 reported to be treated with ICI. Eighty-five percent of respondents were from the United States. The most common conditions noted were thyroid dysfunction in 80 patients, hypertension in 50 patients, chronic kidney disease in 23 patients, heart disease in 10 patients, and diabetes mellitus (DM) in 13 patients. Immune disorders developed in 26 (9%) patients. The limitations are the survey had minimal participation from minority populations. Multiple medical conditions were noted to either emerge or worsen as a result of ICI-based therapies in RCC. Awareness of this information as a starting point should stimulate the development of survivorship programs for renal cancer. A survey of patients with advanced kidney cancer showed that some medical conditions such as thyroid dysfunction, hypertension, heart and kidney disease, DM, and immune conditions were newly diagnosed and/or persisted after immune therapy.

## Introduction

### Survivorship in renal cancer

Immune checkpoint inhibitor (ICI)-based regimens have become standard therapy in multiple malignancies including advanced renal cancer. Renal cancer patients have a chance of long-term remission as noted by the updated survival results of all contemporary immune checkpoint–based combination therapy randomized trials in advanced RCC (1, 2). The combination of ipilimumab and nivolumab showed an overall survival (OS) rate of 30% at a median follow-up of 99 months (3). Median OS was 52.7 months with nivolumab + ipilimumab at the 99 month follow-up timepoint analysis. The 44 month follow-up analysis of CM9ER revealed median OS of 49.5 months with nivolumab plus cabozantinib, translating into a 30% reduction in the risk of death (HR 0.70, 95% CI [0.56, 0.87]) as compared to sunitinib (4). The CLEAR trial showed a survival benefit with median OS of 53.7 months with lenvatinib and pembrolizumab and 54.3 months with sunitinib (5, 6). Around 66.4% of patients were alive at 3 years. At 67 month follow-up analysis of the study KEYNOTE 426, the regimen of axitinib and pembrolizumab demonstrated a 60-month OS rate of 41.9% (7, 8) The four regimens above with the backbone of ICI therapy are FDA approved and are routinely utilized in frontline treatment of metastatic RCC today. The longer follow up for survival of all these studies reveal that prolonged remissions and longer OS in advanced RCC are a reality. Longer follow-up on the studies indicates that a sizeable proportion of patients are living with remission of kidney cancer. Hence, it is of critical importance to recognize the long-term toxicities that may need monitoring and intervention.

### Impact of immune therapy toxicities

The ICI regimens routinely impart toxicities that do not resolve even after discontinuing therapy. There is a wide spectrum of toxicities that can impact multiple systems and involve multiple clinical specialties within medicine. A concerted effort is needed to capture the data around the magnitude of long-term toxicities and their impact on the quality of life, morbidity, and mortality of patients. Keynote 564 has demonstrated benefit in relapse-free survival and OS with adjuvant pembrolizumab in high-risk kidney cancer post nephrectomy (9). Adjuvant therapy with pembrolizumab is FDA approved and has been widely adopted as adjuvant therapy for RCC. Grade 3 or higher adverse events of any cause occurred in 32.4% of the patients who received pembrolizumab and in 17.7% of those who received placebo. The use of adjuvant ICI therapy raises the stakes of tracking life-impacting toxicities as majority of this patient population does not have relapse of cancer and is predicted to have a normal life expectancy. The potential impact on morbidity and mortality should be evaluated in the adjuvant pembrolizumab patient population as well.

To summarize, immune-based regimens have rapidly become the mainstay of frontline therapy in advanced RCC. The median survival is in the range of 48–55 months and long-term remissions are actually feasible. The immune therapy toxicities can potentially be long lasting and life changing. The impact of the therapy on other health conditions has not been studied. With the conversion of advanced RCC to a chronic disease in a subset of patients, awareness and management of these conditions is important. The use of immune checkpoint therapy in the adjuvant setting has made this issue even more critical, as it increased the magnitude of otherwise healthy and asymptomatic patient population getting ICI therapy. We developed a patient perceptions survey questionnaire to evaluate the incidence and the extent of chronic conditions and comorbidities that are occurring or worsening after ICI. This is a report of the results of the survey.

## Methods

The survey was developed in collaboration with the Kidney Cancer Research Alliance (KCCure), with multidisciplinary representation from urologic surgeons, medical oncologists, and patient advocates. The survey was broadcast between July 2022 and September 2022 to patients via website, mailing lists, and social media platforms. The survey was distributed to patients via Facebook, twitter, reddit, via the KCCure website and e-mail distribution list, using unique collectors. Determining an overall denominator across all platforms is not possible. However, the bulk of responses, 69%, was from members of KCCure-administered communities, and the estimated response rate from those communities using reach and click rate analytics was 32%. Multiple responses from the same patient were prohibited via anonymized IP address tracking. Data on the existing pretherapy comorbid conditions were collected, and patient perspective on changes to any medical conditions or any new diagnoses was evaluated in the survey questionnaire.

## Results

### Patient characteristics

**Figure 1:**
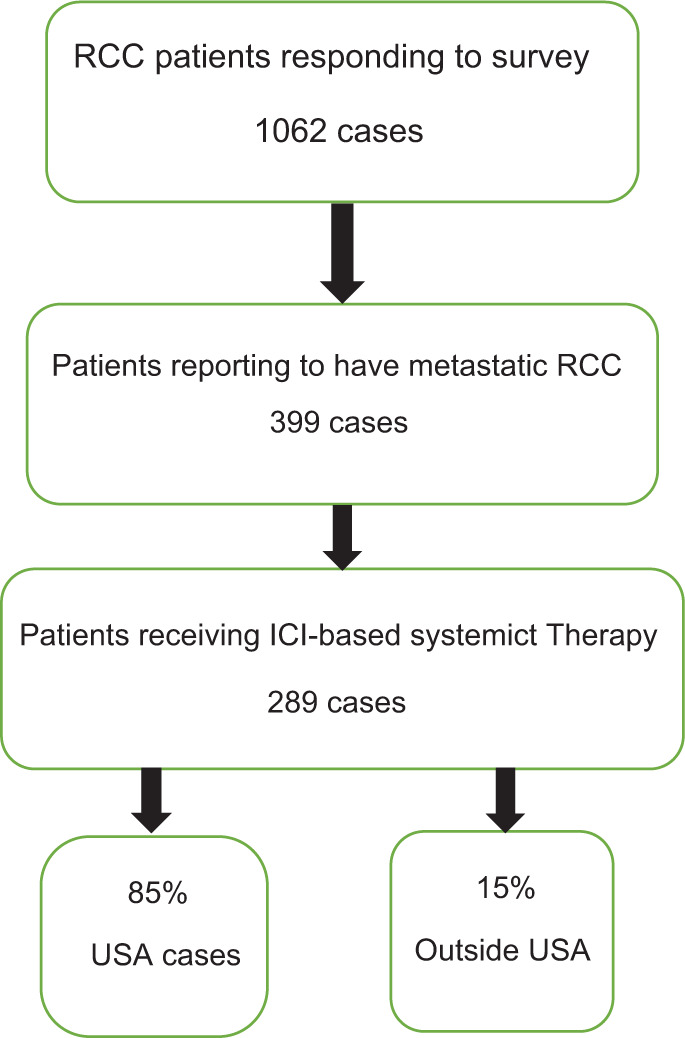
Consort diagram.

**Figure 2:**
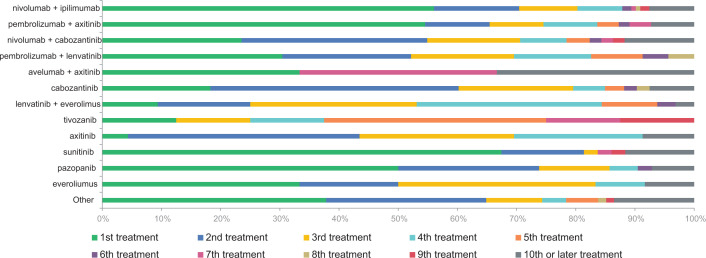
Types and sequence of therapies received.

Patients with metastatic disease being treated with immune checkpoint–based systemic therapy for RCC were included in this survey and analysis. A total of 1062 patients responded to the survey of which 399 patients self-identified as being metastatic and 289 reported to being treated with immune-based systemic therapy (Figure 1). Fifty-six percent of the patients had received ipilimumab and nivolumab combination and the others received a combination of vascular endothelial growth factor tyrosine kinase inhibitor (VEGF-TKI) and programmed death-1 (PD-1) inhibitor (Figure 2). Around 86.85% of patients were from the United States, and 91% were of Caucasian origin. Eleven Hispanic (3.8%), nine Asian (3.1%), and two black (0.69%) patients responded. Around 32.64% of patients were from rural areas. Fifty-three percent were on treatment outside of academic medical centers. Table 1 includes details of patient characteristics. Majority of respondents were from the United States; however, the respondents included patients from all over the world such as nine from Canada; seven from Germany; three each from Philippines, the United Kingdom, and Australia; two each from Denmark, Greece, and India; and one each from South Africa, Malaysia, Brazil, and New Zealand. Two hundred and twenty (76.1%) patients had prior nephrectomy, 199 had radical nephrectomy, and 21 had partial nephrectomy, and 70.4% were cases of clear cell histology.

**Table 1:** Patient characteristics.

Characteristics	Number	Percent
Metastatic Disease and Immune Therapy	289	100
Gender		
Female/Male	143/153	49/51
Race/Ethnicity		
White/Latino/Black/Others	263/11/2/13	91/4/1/4
US/Canada/Germany/Others	251/9/7	87/3/2.4/7.6
Location		
Urban/Suburban/Rural	61/133/94	21/46/33
Treatment Location		
Academic Center/Nonacademic	134/151	47/53
Nephrectomy		
Yes/No/Other Local Therapy	220/48/21	77/16/7
Histology		
Clear/Papillary/Chromophobe/Translocation/Unclassified/Collecting duct/Unknown/Other	195/23/23/7	70/8.3/8.3/2.53
Grade	9/2/16	3.25/0.72/5.78
Grade 4/3/2/1/Unknown	148/40/31/5/69	51.5/13.9/10.8/1.7/24
Sarcomatoid/Rhabdoid/None	45/32/181	18.6/12.9/73.2
Frontline Regimen/Anytime During Therapy		
Ipi+nivo	74/132	25.6/56
Axitinib + pembro	30/55	10.3/54.5
Cabo + nivo	12/51	4.1/23.5
Lenvatinib + pembro	7/23	2.4/30.4
Cabozantinib only	17/93	5.9/18.27
Sunitinib/pazopanib	50/85	17.3/58.6
Other: axitinib, tivozanib, everolimus, Lenvatinib + everolimus, axitinib + avelumab	97	34.4
Immune therapy frontline	124	42.9
Immune therapy second line and beyond	165	57.1
Baseline Medical Conditions		
Present/Absent	161/125	56/44
Htn/DM/Cardiac/Other (preexisting)	125/32/17/44	44/11/6/15
Htn/DM/Cardiac/Other (developed after starting treatment)	50/13/10/147	18/5/4/51
Htn/DM/Cardiac/Other (worsened after treatment)	56/12/10/90	20/5/4/32

### Impact on medical conditions

**Figure 3:**
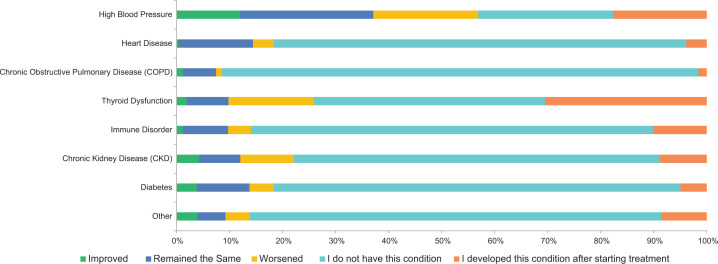
Changes in health conditions post immune based therapy.

One hundred and twenty five patients had no preexisting medical conditions. The baseline medical conditions were predominantly hypertension in 43.7%, followed by diabetes mellitus (DM) and thyroid dysfunction in 11.1% and 10.1%, respectively. Cardiac disease was noted in 5.9% of the patients at baseline. Chronic kidney disease and chronic obstructive pulmonary disease were noted in four (1.4%) and one (0.35%) patient, respectively. The most common condition that developed after starting immune-based therapy was thyroid dysfunction as seen in 81 (30.45%) patients, followed by hypertension in 50 (17.67%) patients, chronic kidney disease in 23 (8.9%) patients, heart disease in 10 (4.9%) patients, and DM in 13 (4.9%) patients . Autoimmune disorders developed in 26 (10.1%) patients (Figure 3 and Table 2). Worsening, and new incidence, of medical conditions were noted after ICI therapy.

**Figure 4:**
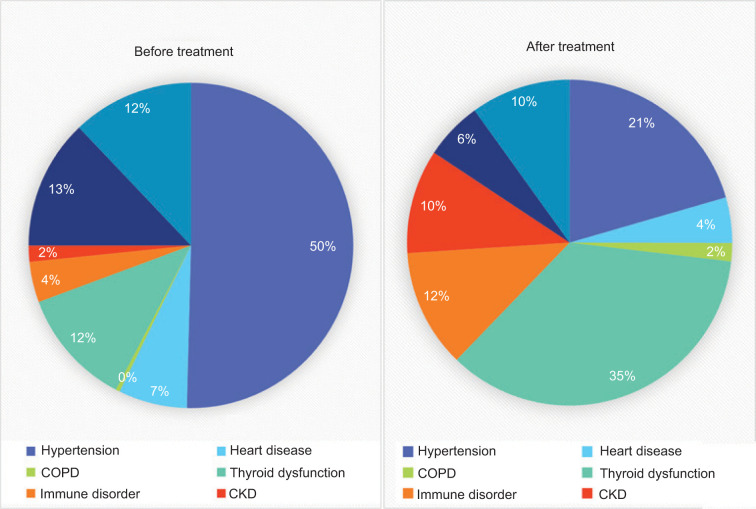
Changes in medical conditions after immune checkpoint therapy.

**Table 2:** Changes in health conditions after immune checkpoint therapy.

	Improved	Remained the same	Worsened	I do not have this condition	I developed this condition after starting treatment	Total
High Blood Pressure	12.01%34	25.09%71	19.79%56	25.44%72	17.67%50	283
Heart Disease	0.39%1	14.01%36	3.89%10	77.82%200	3.89%10	257
Chronic Obstructive Pulmonary Disease (COPD)	1.17%3	6.25%16	1.17%3	89.84%230	1.56%4	256
Thyroid Dysfunction	1.88%5	7.89%21	16.17%43	43.61%116	30.45%81	266
Immune Disorder	1.17%3	8.56%22	4.28%11	75.88%195	10.12%26	257
Chronic Kidney Disease (CKD)	4.26%11	7.75%20	10.08%26	68.99%178	8.91%23	258
Diabetes	3.82%10	9.92%26	4.58%12	76.72%201	4.96%13	262
Other	3.95%6	5.26%8	4.61%7	77.63%118	8.55%13	152

## Discussion

The patient perspectives reported here indicate that immune checkpoint therapy has far-reaching effects. The results of the patient survey indicates the potential for worsening preexisting comorbid conditions, as well as the induction of new medical conditions. Most of these effects are likely to have a lifelong impact and do not reverse even after ICI therapy is discontinued. The patients who were fortunate enough to beat cancer and establish long-term remission will continue to need medical care and follow-up for multiple medical conditions induced by the therapy. In addition, the contribution of therapy-related effects on long-term morbidity and patient mortality need to be evaluated.

To our knowledge, this is the first patient-reported outcomes study questionnaire gauging the chronic effects of ICI in RCC. There are multiple reports of chronic irreversible toxicities induced by ICI in other malignancies such as melanoma and lung but not specifically in RCC (10–16). The significance of this global survey is that patient-reported outcomes are a powerful tool to evaluate the impact on the quality of life. Provider assessments frequently underreport the chronic toxicities and focus only on the long-term efficacy of ICI. Patients are likely in the best position to judge the comorbid conditions they have acquired or the conditions that have worsened, related to ICI therapy and the impact thereof on their daily lives. We realize that some of these delayed toxicities overlap with and may be induced by the VEGF-TKI therapies, but many of those are reversible when the agent is discontinued, unlike the ICI therapies. However, since combinations are typically used in RCC, the overall toxicity impact needs to be considered. This study establishes a baseline for the far-reaching influence of immune checkpoint-based regimens on the lives of patients with RCC.

The wide spectrum of medical problems are managed in distinct subspecialty clinics making it challenging to evaluate an overall impact. The patient questionnaire enables us to receive information of individual chronic toxicities and medical conditions that are impacting RCC survivors. Phase III trials are not designed or equipped to handle the long-term and detailed follow-up required for this, especially in patients who have discontinued study therapy because of either toxicity or remarkable efficacy. The value of this study lies in the fact that it shines a light on the medical conditions induced or worsened by ICI and creates an awareness of this problem. It provides preliminary data that will help guide the development of future interventions.

We do realize that the study has some limitations. The timepoints of the chronic conditions and the severity were not assessed by the survey. The rate of early mortality or impact on life expectancy has not been measured. The survey had underrepresentation of minorities where the incidence of comorbid medical conditions is likely to be higher. It would be important to do a focused survey specific to minority patient populations. Overall, the survey served as a tool to highlight the patient perspectives on long-term toxicities from ICI therapy.

From a clinical trial planning perspective, it is important to consider cancer-specific and overall survival as distinct endpoints. Conventionally, for metastatic solid tumor trials, overall survival has been considered the gold standard for demonstrating effects of a therapeutic intervention. However, the increasing magnitude of chronic toxicities imparted by ICI highlights the need to evaluate the impact of the toxicities on life expectancy. Multiple groups have recognized this and reported the incidence and chronic impact of immune toxicities. A retrospective six center study of advanced melanoma patients has reported that chronic immune-related adverse events persisted in 46.2% of which 50.3% were grade 2 or higher (10). Majority of the patients (68.0%) were symptomatic. Around 36.7% experienced resolution of chronic effects with median time to resolution of 19.7 [14.4–31.5] months from ICI start and of 11.2 months from discontinuing therapy. Among patients with persistent effects, 25% required systemic steroids. The most common persistent ICI effects were hypothyroidism, which is concordant with that noted in our patient questionnaire. The others were arthritis, dermatitis, and adrenal insufficiency in 33.3%, 16.7%, and 14.8%, respectively (11). A Pubmed and EMBASE search for nonendocrine chronic toxicities revealed 323 patients from 225 studies with primarily melanoma and lung cancer (12). The chronic irAEs experienced were rheumatological in 20% of patients, followed by neurological in 19%, gastrointestinal in 16%, and dermatological in 14%. Thirty percent of patients had ongoing symptoms in the past 6 months, and 76% required oral and/or intravenous steroids (12, 13).

The time has arrived for establishing immune therapy survivorship clinics. These would need to be multispecialty clinics as the conditions span multiple clinical systems and require specialized therapeutic and prophylactic interventions. It remains an ongoing challenge to address these conditions in an average oncology clinic, which is geared toward assessing clinical symptoms, physical examination, and reviewing imaging, with the goals of early detection and therapy planning of cancer relapse. The incremental efficacy and expanding use of immune therapies in oncology has created the need for a multidisciplinary survivorship clinic to address the unique needs of patients with chronic toxicities.

## Conclusion

There is a clinically significant incidence of medical conditions emerging as a result of immune checkpoint inhibitor therapies in RCC. This information would be a starting point for studying the long-term effects of immune therapy and would lead toward development of survivorship programs for renal cancer.
